# Larvicidal activity of *Syzygium aromaticum* (L.) Merr and *Citrus sinensis* (L.) Osbeck essential oils and their antagonistic effects with temephos in resistant populations of *Aedes aegypti*

**DOI:** 10.1590/0074-02760160075

**Published:** 2016-07-04

**Authors:** Adriana Faraco de Oliveira Araujo, João Tadeu Ribeiro-Paes, Juliana Telles de Deus, Sócrates Cabral de Holanda Cavalcanti, Rogéria de Souza Nunes, Péricles Barreto Alves, Maria de Lourdes da Graça Macoris

**Affiliations:** 1 Universidade Estadual Paulista, Faculdade de Ciências e Letras, Departamento de Ciências Biológicas, Assis, SP, Brasil; 2 Superintendência de Controle de Endemias, Marília, SP, Brasil; 3 Universidade Federal de Sergipe, Departamento de Farmácia, São Cristóvão, SE, Brasil; 4 Universidade Federal de Sergipe, Departamento de Química, São Cristóvão, SE, Brasil

**Keywords:** Syzygium aromaticum, Citrus sinensis, Aedes aegypti, vector control, larvicide, dengue

## Abstract

Environmentally friendly botanical larvicides are commonly considered as an alternative to synthetic larvicides against *Aedes aegypti* Linn. In addition, mosquito resistance to currently used larvicides has motivated research to find new compounds acting via different mechanisms of action, with the goal of controlling the spread of mosquitos. Essential oils have been widely studied for this purpose. This work aims to evaluate the larvicidal potential of *Syzygium aromaticum* and *Citrus sinensis* essential oils, either alone or in combination with temephos, on *Ae. aegypti* populations having different levels of organophosphate resistance. The 50% lethal concentration (LC50) of the essential oils alone and in combination with temephos and the influence of essential oils on vector oviposition were evaluated. The results revealed that essential oils exhibited similar larvicidal activity in resistant populations and susceptible populations. However, *S. aromaticum* and *C. sinensis* essential oils in combination with temephos did not decrease resistance profiles. The presence of the evaluated essential oils in oviposition sites significantly decreased the number of eggs compared to sites with tap water. Therefore, the evaluated essential oils are suitable for use in mosquito resistance management, whereas their combinations with temephos are not recommended. Additionally, repellency should be considered during formulation development to avoid mosquito deterrence.

Dengue is currently considered to be one of the most important arboviroses, accounting for substantial morbidity and mortality worldwide. This virus represents a serious public threat, especially in tropical countries, where the warm temperature and environmental conditions favour the development and proliferation of its disease vector, *Aedes aegypti* Linn. (Sharp et al. 2014). Furthermore, there is currently no vaccine available that acts against the different virus serotypes. As a result, prevention efforts are restricted to focusing exclusively on mosquito control, and the interventions available for prevention and control actions in combatting the vector are therefore limited (Bisset et al. 2011, Coelho 2012). Control strategies frequently focus on the use of chemical larvicides and insecticides, which are mainly organophosphates, such as temephos, and pyrethroids. Such control actions are integrated with environmental management. However, the frequent use of such products has selected for mosquito populations that are resistant to these chemicals (Pocquet et al. 2014). In addition, the environmental impacts of pesticides have been widely studied, as they are responsible for non-target toxicity and water stream contamination (Muhammetoglu et al. 2010).

Resistance is commonly related to the activity of enzymes present in the mosquito biochemical routes of insecticide metabolism. Resistance data from São Paulo state reports that the major pathway for the detoxification of temephos, the larvicide that has been widely used in Brazil for over 30 years, is performed by esterases (Melo-Santos et al. 2010). Given the characteristics of *Ae. aegypti* populations that are resistant to temephos and other larvicides, the demand for alternative plant-based products with a different mode of action than that which conferred organophosphate resistance is necessary.

In recent years, numerous investigations have been conducted in the search for active compounds from natural sources, with an emphasis on medicinal and aromatic plant essential oils. These oils are widely known for their larvicidal properties, as well as antiviral, fungicidal, antiparasitic and cosmetic properties (Palazzolo et al. 2013, Mittal et al. 2014). The vast Brazilian biodiversity and the insecticidal potential of several unexplored species may provide alternative novel chemical agents to control culicids, especially *Ae. aegypti* (Brandão et al. 2013, Novaes et al. 2013). The use of plants-based chemicals as an alternative for vector control is based on their usually low toxicity to animals and aquatic ecosystems, in addition to their biodegradability and environmental safety. In contrast, synthetic insecticides, which can cause resistance, are toxic and pollutants (Corrêa & Salgado 2011).

Several studies have demonstrated the activity of *Syzygium aromaticum* (L.) Merr. & Perry and *Citrus sinensis* (L.) Osbeck essential oils, as well as their major components, against *Ae. aegypti* larvae (Cavalcanti et al. 2004, Costa et al. 2005, Barbosa et al. 2012, Murugan et al. 2012, Warikoo et al. 2012, Fayemiwo et al. 2014). In this context, we aimed to further evaluate the larvicidal activity of *S. aromaticum* and *C. sinensis* essential oils on insecticide-resistant *Ae. aegypti* populations, as well as to assess the combined effects of these essential oils and temephos. Furthermore, the oviposition behaviour of females in the presence of these larvicidal agents was evaluated because repellence is detrimental to larvicidal action.

## MATERIALS AND METHODS

*Chemicals and essential oil extraction* - Temephos (97.5%) and Tween 80 were purchased from Sigma-Aldrich Chemical Co. (St. Louis, MO, USA). Sodium sulphate was purchased from Synth (Brazil). The *C. sinensis* fruits and dry clove buds (Maratá^TM^, Itaporanga D’Ajuda, SE, Brazil) used in this study were acquired from a local market in Aracaju city, Sergipe state, Brazil, in a single lot. *C. sinensis* peels were dried in a circulating air oven at 40ºC for three days. The dry materials were separately ground and submitted to hydrodistillation for 3 h in a Clevenger-type apparatus to yield yellowish oils. The essential oils obtained were separated from the aqueous phase, followed by the addition of sodium sulphate (Na_2_SO_4_) to remove water residue, and were then filtered and kept in a freezer until further analysis and activity evaluation.

*Analytical conditions* - The essential oils obtained by hydrodistillation were analysed by gas chromatography-mass spectrometry (GC-MS) using a Shimadzu QP5050A (Shimadzu Europe, North Rhine-Westphalia, Germany) gas chromatograph equipped with a DB-5 MS fused silica column (30 m x 0.25 mm; film thickness 0.25 μm), under the following conditions: helium as the carrier gas at 1.0 mL/min; injector split at 250ºC (split ratio 1/20); detector at 280ºC; column temperature programme of 80ºC for 1.5 min, increase of 4ºC per min to 180ºC, then 10ºC per min to 300ºC, ending with a 10 min isothermal at 300ºC. The mass spectra were taken at 70 eV with a scanning speed of 0.85 scan/s from 40 to 550 Da. Peak identification was made on the basis of comparison of their retention indices relative to an *n*-alkane homologous series obtained by co-injecting the oil sample with a linear hydrocarbon mixture.

Quantitative analysis of the chemical constituents was performed by flame ionization gas chromatography (FID) using a Shimadzu GC-17A (Shimadzu Corporation, Kyoto, Japan) instrument under the following operational conditions: a capillary ZB-5MS column (5% phenyl-arylene-95%-dimethylpolysiloxane) fused silica capillary column (30 m x 0.25 mm i.d. x 0.25 μm film thickness) from Phenomenex (Torrance, CA, USA), under the same conditions reported for the GC-MS. Quantification of each constituent was estimated by area normalisation (%). The compound concentrations were calculated from the GC peak areas and arranged in the order of GC elution.

*Stock solutions* - Mixtures of *C. sinensis* or *S. aromaticum* essential oils and temephos at ratios of 54:1 and 100:1, respectively, were prepared based on previous LC_50_ data for both essential oils and temephos separately. Thus, a 1 L solution containing 55 mg of *C. sinensis*/temephos or 101 mg of *S. aromaticum/*temephos would contain a concentration approximately equivalent to the LC_50_ of the essential oil and 1 ppm of temephos.

A 20,000 ppm stock solution was prepared using each essential oil or its mixture with temephos (20 mg/mL), Tween 80 (5 mL), and deionized water (1 mL). The stock solution was used to make 150 mL water solutions ranging from 0.1 to 220 ppm. Temephos was also separately used to monitor *Ae. aegypti* susceptibility.

*Mosquitoes* - The Rockefeller strain of *Ae. aegypti* was maintained under laboratory conditions at a controlled temperature of 25ºC ± 2ºC and a relative humidity of 70 ± 10%. Insecticide-resistant populations were collected from the following counties: Marília, Araçatuba and Santos, São Paulo state, Brazil. Resistant populations were maintained under the same conditions as the Rockefeller strain but in separate rooms. Populations were selected based on previously evaluate levels of insecticide-detoxificant enzymes (α- and β-esterases and glutathione-S-transferase). Additionally, the levels of multi-function oxidase enzyme activity in the Marília population were considered normal, while the Araçatuba and Santos populations presented altered activity (Macoris et al. 2003).

*Larvicidal assay* - Assays were conducted at the Applied Entomology Laboratory (SUCEN, Marília, São Paulo, Brazil) under a controlled environment and were performed according to the procedure recommended by the World Health Organization (WHO 2005). Concentration ranges were determined using a previously generated concentration-response curve for 20 third-instar larvae. Eight different concentrations and four replicates were used for each essential oil mixture and the control. Twenty late third-instar larvae were exposed to 150 mL solutions of larvicide in disposable cups. A mortality count was conducted 24 h after treatment. Each experiment was repeated three times in different days, in such a way that 240 larvae were exposed to each larvicide concentration or controls. The controls were prepared with Tween 80 and water at the highest concentration used in each experiment. The organophosphate temephos, a standard insecticide for larvae control, was used as a positive control. In all cases where mortality of between 5-20% occurred in the control experiment, the data were corrected using Abbott’s formula (% deaths = [1-(test/control)] x 100) (Abbott 1925). Any experiment with over 20% mortality in controls was discarded.

*Oviposition behaviour* - To evaluate whether the presence of essential oils in oviposition sites influence the oviposition behaviour of females, two rearing cages, one for each essential oil, were set with field-collected (Marília, São Paulo, Brazil), newly emerged *Ae. aegypti* (100 females and 50 males). The mosquitoes were fed with 10% honey in cotton pads. After a three-day long copulation period, the cotton pads were removed. On the fourth day, blood was offered via an artificial membrane blood-feeding apparatus. Honey and blood feeding were then offered every other day until the end of the experiment, which extended for 33 days.

Three days after blood feeding, two sites for oviposition were placed in each rearing cage to monitor possible oviposition preferences. One site consisted of a disposable cup containing tap water, and a second cup contained essential oil. The cups were lined with filter paper as a substrate for oviposition and were covered with black cups to control the availability of light. The essential oil concentrations of *C. sinensis* and *S. aromaticum* were 81.44 and 860 ppm, respectively, approximately four times the LC_99_ for the Rockefeller strain, an arbitrary criterion for the use of larvicides in the field (WHO 2005).

Blood feeding was offered three times a week (Mondays, Wednesdays and Fridays). At these times, the paper filters were removed and allowed to dry, and the larvicidal solutions were replaced. To prevent possible effects of local brightness, which can interfere with the choice of oviposition site, the cups were also repositioned during this procedure. After 24 h, the number of eggs was quantified.

*Statistical analysis* - Probit analysis was conducted on the mortality data collected after 24 h of exposure to different concentrations of the testing solutions to establish lethal concentrations (LC_50_, LC_95_, and LC_99_) and 95% confidence intervals (CI) for the respective essential oils or mixtures using the Polo-PC software suite (LeOra Software 1987). The activity of the essential oils or mixtures were considered to be significantly different when their 95% CIs failed to overlap. The lethal concentrations were used to calculate the resistance ratio (RR) using Equation 1.


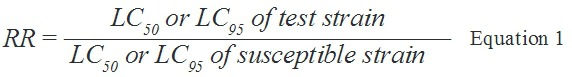


The concentration-response graphics were created using Graph Pad Prism software version 6.0.

The oviposition index (OI, Equation 2) was used to evaluate the oviposition response of females to different sites of oviposition (Marques et al. 2013). The OI varies between -1 and +1. Substances attracting or stimulating egg deposition result in a positive OI, while substances repelling or inhibiting egg deposition result in a negative OI.


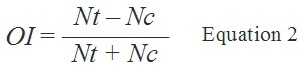


Where OI is the oviposition index, Nt is the average number of eggs in the test solution and Nc is the average number of eggs in the control solution.

Student’s t-test was used to compare the number of oviposited eggs in each oviposition site. Graphs showing the total number of eggs in each oviposition site and the total number of eggs over the course of the experiment were created using Graph Pad Prism software version 6.0.

## RESULTS AND DISCUSSION

The essential oils of *C. sinensis* and *S. aromaticum* were obtained at 9.1-19.0% yield, respectively. Four compounds were identified in the essential oil of *C. sinensis*, representing 99% of the essential oil, while five compounds were identified in the essential oil of *S. aromaticum*, representing 100% of the essential oil. Their retention indices and composition percentages, listed in the order of elution in the ZB-5MS column, are given in Table I.

**TABLE I:** Essential oil compositions of *Citrus sinensis* and *Syzygium aromaticum*

RI	Compound	*C. sinensis* (% FID)	*S. aromaticum* (% FID)
998	Mircene	1.27	-
1003	*n*-Octanol	1.09	-
1028	Limonene	91.88	-
1099	Linalool	4.76	-
1354	Eugenol	-	65.99
1421	b-Caryophyllene	-	28.32
1455	α-humulene	-	2.34
1522	Eugenol acetate	-	3.35

Total		99.00	100.00

FID: flame ionization gas chromatography; RI: relative retention index calculated against *n*-alkanes, applying the Van den Dool equation; %: compound percentage.

The major component of *C. sinensis* was identified as limonene, accounting for 91.88% of the essential oil, and for *S. aromaticum*, the main component was eugenol, accounting for 65.99% of the essential oil.

The *Ae. aegypti* larvae susceptibility assays demonstrated that *S. aromaticum* and *C. sinensis* essential oils have larvicidal effects in all tested populations. Because no mortality above 20% in the control groups was observed, the use of Tween 80 as a surfactant had no effect on mortality; therefore, none of the assays were invalidated. Lethal concentrations and their respective CIs are presented in Table II.

**TABLE II:** Lethal concentrations (LC_50_, LC_95_, and LC_99_) and confidence intervals (CI) of essential oils and their combinations with temephos in *Aedes aegypti* field-collected in Araçatuba, Marília and Santos and the Rockefeller strain

Larvicide	Strain	LC_50_ (95% CI) ppm	LC_95_ (95% CI) ppm	LC_99_ (95% CI) ppm
*Citrus sinensis*	Rockefeller	11.92 (11.7 to 12.2)	17.40 (16.4 to 18.9)	20.36 (18.8 to 22.7)
	Araçatuba	15.06 (14.7 to 15.5)	27.59 (26.3 to 29.1)	35.45(33.3 to 38.1)
	Marília	13.70 (13.4 to 14.0)	21.08 (20.4 to 21.9)	25.20 (24.1 to 26.6)
	Santos	16.30 (15.5 to 17.1)	34.18 (31.5 to 37.7)	46.40 (41.7 to 53)
*Syzygium aromaticum*	Rockefeller	93.56 (90.1 to 97.0)	167.85 (157.2 to 182.1)	213.83 (195.6 to 239.0)
	Araçatuba	92.97 (90.0 to 95.8)	163.40 (156.2 to 172.0)	206.40 (194.4 to 221.4)
	Marília	106.90 (103.9 to 109.8)	174.20 (167.7 to 181.9)	213.20 (202.6 to 226.3)
	Santos	95.00 (92.2 to 97.8)	155.50 (148.3 to 164.4)	190.70 (179.1 to 205.6)
*C. sinensis* + temephos	Rockefeller	0.33 (0.29 to 0.35)	0.57 (0.53 to 0.65)	0.73 (0.64 to 0.88)
	Araçatuba	1.65 (1.48 to 1.81)	5.13 (4.61 to 5.83)	8.21 (7.08 to 9.86)
	Marília	1.78 (1.61 to 1.95)	5.48 (4.39 to 7.80)	8.75 (6.43 to 14.33)
	Santos	1.88 (1.65 to 2.09)	5.35 (4.62 to 6.56)	8.25 (6.70 to 11.14)
*S. aromaticum* + temephos	Rockefeller	0.72 (0.69 to 0.75)	1.00 (0.94 to 1.08)	1.14 (1.06 to 1.26)
	Araçatuba	1.66 (1.5 to 1.8)	4.39 (3.8 to 5.4)	6.60 (5.36 to 8.81)
	Marília	1.87 (1.41 to 2.21)	5.20 (4.21 to 7.74)	7.94 (10.98 to 24.13)
	Santos	1.96 (1.75 to 2.14)	3.96 (3.53 to 4.65)	5.30 (4.54 to 6.66)
Temephos	Rockefeller	0.0035 (0.0034 to 0.0036)	0.0052 (0.0050 to 0.0054)	0.0061 (0.0058 to 0.0065)
	Araçatuba	0.0130 (0.012 to 0.013)	0.0240 (0.023 to 0.026)	0.0310 (0.028 to 0.034)
	Marília	0.0088 (0.0083 to 0.0092)	0.0180 (0.017 to 0.019)	0.02400 (0.022 to 0.027)
	Santos	0.0120 (0.011 to 0.012)	0.0200 (0.0191 to 0.022)	0.0260 (0.024 to 0.028)

95% CI: ninety-five percent probability confidence interval.

*C. sinensis* evaluated as a larvicide exhibited significantly different LC_95_s in different *Ae. aegypti* populations because no overlap in 95% confidence intervals was observed for any population. In contrast, the LC_95_ for *S. aromaticum* exhibited no significant differences between the field-collected populations and the Rockefeller strain, and within the field-collected populations, only the LC_95_s for the Santos and Marília populations were significantly different. The average CI ranges for *C. sinensis* and *S. aromaticum* were 3.25 and 17.75, respectively, demonstrating a greater variability of the latter because it had verified activity over a larger range of concentrations.

The combinations of essential oils and temephos evaluated in the field-collected populations of *Ae. aegypti* had a significantly different LC_95_ compared to that of the Rockefeller strain, exhibiting a resistance profile close to that of temephos alone.

Chaieb et al. (2007) and Simas et al. (2004) found that LC_50_ = 44.5 ppm for eugenol, the major compound in *S. aromaticum* essential oil, in *Ae. aegypti* populations. The larvicidal activity of *S. aromaticum* essential oil has been previously reported by several authors. Costa et al. (2005) found that LC_50_ = 21.4 ppm , while Fayemiwo et al. (2014) found LC_50_ = 92.56 and Barbosa et al. (2012) obtained values of 62.3 and 77.0 ppm for field-collected and Rockefeller larvae, respectively. The differences in the LC_50_ values observed in the present and previous studies can be explained as a result the use of different populations, methodologies, and the composition of the essential oils tested.

The pH of the deionized water was not altered by the addition of essential oils or mixtures, even at the highest concentration used in the study, eliminating the possibility of larvae mortality caused by increased acidity.

According to Mazzari and Georghiou (Mazzarri & Georghiou 1995), a mosquito strain is considered susceptible when its resistance ratio is less than five, moderately resistant when the resistance ratio is between five and ten, and highly resistant when the resistance ratio is greater than ten. Accordingly, the results of this study show that the resistance levels to the essential oils alone are low and the field-collected strains are susceptible to the composition of the essential oils. However, the resistance ratio for temephos and the essential oil/temephos combinations is considered moderate (Table III). The *C. sinensis*/temephos combinations exhibited resistance ratios close to ten. Therefore, within the evaluated products, the *C. sinensis*/temephos mixture is less suitable for use as a larvicide to control this vector.

**TABLE III:** Resistance ratios to the larvicidal compositions for resistant strains

Larvicide	Strain	RR LC_50_	RR LC_95_	RR LC_99_
*Citrus sinensis*	Araçatuba	1.3	1.6	1.7
	Marília	1.1	1.2	1.2
	Santos	1.4	2.0	2.3
*Syzygium aromaticum*	Araçatuba	1.0	1.0	1.0
	Marília	1.1	1.0	1.0
	Santos	1.0	0.9	0.9
Temephos	Araçatuba	3.7	4.6	5.1
	Marília	2.5	3.5	3.9
	Santos	3.4	3.8	4.3
*C. sinensis* + temephos	Araçatuba	5.0	9.0	11.2
	Marília	5.4	9.6	12.0
	Santos	5.7	9.4	11.3
*S. aromaticum* + temephos	Araçatuba	2.3	4.4	5.8
	Marília	2.6	5.2	7.0
	Santos	2.7	4.0	4.6

The Brazilian *Ae. aegypti* insecticide resistance monitoring network (MoReNAa) suggests a substitution of the larvicide when the resistance ratio is equal to or higher than three (Secretaria de Vigilância em Saúde 2006, unpublished data). Therefore, *C. sinensis* and *S. aromaticum* essential oils are potential candidates for use as auxiliary larvicides to control *Ae. aegypti* strains that are resistant to temephos, while their combinations with temephos are not recommended for use as larvicides.

**Fig. 1:**
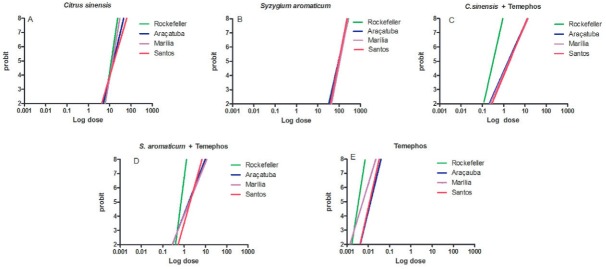
: dose-response curves created using bioassay data after exposure to the essential oils of *Citrus sinensis* (A) and *Syzygium aromaticum* (B), temephos combinations (C-D) and temephos alone (E).

Concentration-response plots were generated based on the methodology proposed by Bliss (1935) and Fisher (1935), which allows for the comparison between different populations and tested products by transforming the sigmoid concentration-response curves to lines by converting the concentration to a logarithmic scale and the percent mortality to a probit scale, which ranges from two-eight. The lines are illustrated in Fig. 1.

The concentration required by *S. aromaticum* to act as a larvicide is approximately 10 times higher than that of *C. sinensis* (Fig. 1, plots 1A-B). However, the mortality response obtained by the former essential oil in all populations is more homogeneous than that of *C. sinensis*. Furthermore, the concentration needed for temephos alone to act as a larvicide is lower than that of the essential oil/temephos combinations (Fig. 1, plots 1C-E).

According to the concept proposed by Brindley and Selim (Brindley & Selim 1984), synergists are substances that, although used in sublethal concentrations, confer a higher mortality than that of each single agent. The concentration of temephos to achieve the LC_50_ in the temephos/essential oil mixtures was higher than that of temephos alone for all tested populations. Therefore, the essential oils in combination with temephos exerted an antagonistic effect (Table IV).

**Fig. 2:**
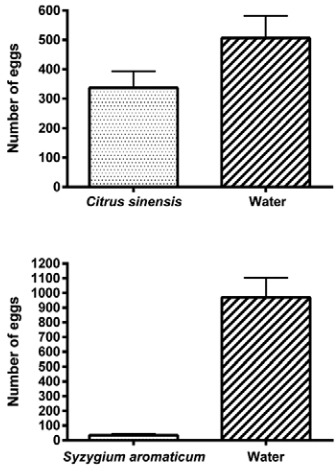
: total number of eggs laid in paired oviposition sites, with and without the presence of each essential oil.

The results of the oviposition behaviour assay demonstrated that gravid females oviposited in all available sites. However, the number of eggs in each site was significantly different (p < 0.05). The number of eggs at oviposition sites containing essential oils was significantly lower than the sites containing tap water, as described in Table V and illustrated in Fig. 2.

**TABLE IV:** Concentration of temephos at LC_50_ for the essential oil/temephos combinations and temephos alone

Strain	Concentration of temephos^1^	Concentration of temephos^2^	Concentration of temephos^3^
Rockefeller	0.004	0.006	0.007
Araçatuba	0.013	1.620	0.016
Marília	0.009	1.748	0.019
Santos	0.012	1.846	0.019

^1^alone; ^2^in combination with *Citrus sinensis*; ^3^in combination with *Syzygium aromaticum*.

**TABLE V:** Oviposition response of female *Aedes aegypti* in oviposition sites containing water, *Citrus sinensis*, and *Syzygium aromaticum* essential oils

	*S. aromaticum*	Water	*C. sinensis*	Water
Total number of eggs *per* oviposition site	515	14536	5050	7592
Average number of eggs every 48 h	34	969	337	506
Standard deviation	40.98	521.49	220.61	295,35
Student’s t-test	0.000002	-	0.0006	-
Oviposition Index	-0.93	-	-0.2	-

Marques et al. (2013) reported the oviposition index (OI), ranging from -1 to +1, to evaluate oviposition-attractant or -repellent substances. A positive OI means that the substance has an attractant activity, while a negative value means the substance has a repellent activity. *C. sinensis* and *S. aromaticum* exhibited OIs of -0.2 and -0.93, respectively. Although *C. sinensis* exhibited a low repellency profile, the essential oils evaluated here exhibit negative OIs and therefore have repellent effects on *Ae. aegypti* oviposition. According to Machado and Fernandes Jr (2011), essential oils are natural, volatile and complex compounds, characterised by a strong odour, which influence the ovipositional behaviour of the gravid female. Repellence is an aggravating influence for larvicides because the vector avoids oviposition in treated containers, favouring non-treated water sources for oviposition. These results should be considered in the development of new formulations because essential oil volatility increases repellency, and therefore low volatile formulations will decrease repellency.

*C. sinensis* and *S. aromaticum* exhibit similar larvicidal activities in *Ae. aegypti* populations with different susceptibility profiles to the organophosphate temephos. Therefore, the evaluated essential oils have the potential to be used in mosquito control as an alternative to overcome *Ae. aegypti* resistance.

The mechanism of action of the essential oil of *S. aromaticum* is most likely different from that of temephos because no significant difference was observed between the larvicidal activity for this essential oil in susceptible Rockefeller and resistant strains. In contrast, *C. sinensis* essential oil exhibited significantly different larvicidal activities between the evaluated populations, which indicates either cross-resistance to previously used larvicides or close contact to this essential oil, as São Paulo state is the main citrus producer in Brazil.

The combination of the evaluated essential oils and temephos did not display increased toxicity; therefore, no synergistic effect was observed. Additionally, resistance was found to essential oil/temephos combinations, and therefore the combined use of the evaluated essential oils and temephos is not recommended.

The essential oils evaluated here exhibit repellent effects on the oviposition behaviour of *Ae. aegypti* females. Therefore, volatility should be considered during the development of larvicides formulations containing these products, with the goal of reducing volatility and consequently reducing repellency.

The results suggest that the *S. aromaticum* and *C. sinensis* essential oils represent an alternative as low-toxicity natural larvicides and a promising approach for *Ae. aegypti* control, especially in strains showing resistance to currently employed larvicides, such as organophosphates.
